# *Nras* Overexpression Results in Granulocytosis, T-Cell Expansion and Early Lethality in Mice

**DOI:** 10.1371/journal.pone.0042216

**Published:** 2012-08-02

**Authors:** Louise Berkhoudt Lassen, Borja Ballarín-González, Alexander Schmitz, Annette Füchtbauer, Finn Skou Pedersen, Ernst-Martin Füchtbauer

**Affiliations:** 1 Department of Molecular Biology and Genetics, Aarhus University, Aarhus, Denmark; 2 Department of Haematology, Aalborg Hospital, Aarhus University Hospital, Denmark; University of Saarland Medical School, Germany

## Abstract

*NRAS* is a proto-oncogene involved in numerous myeloid malignancies. Here, we report on a mouse line bearing a single retroviral long terminal repeat inserted into *Nras*. This genetic modification resulted in an increased level of wild type *Nras* mRNA giving the possibility of studying the function and activation of wild type NRAS. Flow cytometry was used to show a variable but significant increase of immature myeloid cells in spleen and thymus, and of T-cells in the spleen. At an age of one week, homozygous mice began to retard compared to their wild type and heterozygous littermates. Two weeks after birth, animals started to progressively lose weight and die before weaning. Heterozygous mice showed a moderate increase of T-cells and granulocytes but survived to adulthood and were fertile. In homozygous and heterozygous mice *Gfi1* and *Gcsf* mRNA levels were upregulated, possibly explaining the increment in immature myeloid cells detected in these mice. The short latency period indicates that *Nras* overexpression alone is sufficient to cause dose-dependent granulocytosis and T-cell expansion.

## Introduction

RAS proteins constitute a family of signal-transducing GTPases involved in many basic cellular processes such as cell cycle progression and apoptosis. The inherent GTPase activity of RAS is controlled by guanine nucleotide exchange factors (GEFs) and GTPase-activating proteins (GAPs), which promote the shuttling between the active (GTP bound) and inactive (GDP bound) states of the RAS proteins. Spatial and temporal activation of RAS proteins is tightly regulated [1], [2] and aberrant RAS signaling can lead to congenital developmental disorders [3] and oncogenic transformation. More than 15% of all human tumors contain activating mutations of the *NRAS*, *KRA*S, or *HRAS* homologs (reviewed in [4]) and in many other cases, overexpression or hyper-activation of the wild type protein has been described (reviewed in [5]).

Despite the high similarity among the RAS proteins, the aberrant expression of the different homologs is associated with particular types of human cancer [6]. Additional evidence for differential functions of RAS homologs has been provided by genetically modified mouse models. While homozygous *Nras* and *Hras* single and double knock-out (KO) mice are viable and reproduce normally, *Kras* homozygous KO mice die during embryonic development [7], [8], [9], [10]. If the *Kras* coding sequence is replaced by *Hras*, the embryonic lethality is rescued but adult animals develop dilated cardiomyopathy and elevated blood pressure, suggesting a unique role of KRAS in cardiovascular homeostasis [11].

Homozygous *Nras* KO mice are overall healthy, but present impaired antiviral immune response and T-cell function due to a reduced population of CD8^+^ cells in the thymus. After influenza virus infection, *Nras* KO mice showed a reduced response of CD4^+^ T lymphocytes, granulocytes, NK cells, macrophages, and CD8^+^ T lymphocytes. Notably the overall levels of RAS proteins in *Nras* KO mice remain unchanged due to a compensatory increase of KRAS and HRAS, suggesting a specific role for NRAS in lymphoid cells [12].

The role of elevated RAS in cancer has been investigated in several mouse models. Myeloid malignancies with incomplete penetrance and long latency periods were observed when the bone marrow of irradiated mice was repopulated with cells overexpressing a constitutively active NRAS (NRAS^G12D^) protein. Several animals presented increased numbers of granulocytes at the expense of lymphocytes, but due to the long latency and low penetrance, it was suggested that a secondary hit is required to induce cancer [13]. However, when NRAS^G12D^ was expressed in early hematopoietic cells from an MSCV retroviral vector, myeloid disorders resembling human AML and CMML were efficiently induced by higher and lower NRAS signaling levels respectively [14].

Likewise, expression of constitutively active mutants of HRAS and KRAS (HRAS^G12V^ and KRAS^G12D^) also showed dose dependent induction of AML- or CMML-like diseases [15].

Heterozygous expression of NRAS^G12D^ from the endogenous *Nras* locus in epiblast cells results in embryonic lethality [16]. Heterozygous expression only in liver, spleen and bone marrow results in a mild phenotype dependent on genetic background and characterized by a wide spectrum of hematologic diseases [16], [17]. However, upon infection with the MOL4070LTR retrovirus, these animals developed AML considerably faster than control mice [17]. Homozygous expression leads to myeloid hyperplasia with shorter latency [16]. In semisolid culture, the bone marrow cells displayed an abnormal growth pattern, and after co-transplantation with competitor wild type cells into irradiated recipient mice, a dose-dependent phenotype was observed. Almost all animals with heterozygous expression of *Nras* ^G12D^ developed a CMML-like disease starting after 6 months. The mice suffered from anemia and developed myeloid hyperplasia in bone marrow and spleen [18]. Homozygous expression did not induce sustained CMML but resulted in acute T-cell lymphoblastic leukemia/lymphoma [16]. Increment of the number of transplanted cells changed the disease pattern of the recipient mice, as lethal myeloproliferative disease was induced in the majority of the mice independent on the number of *Nras* ^G12D^ alleles. Incidences resembling human B- and T-cell lymphoblastic leukemia/lymphoma were likewise observed but only upon transplantation of *Nras* ^G12D/G12D^ cells [19].

An *Nras^G12D^hypo* allele expressed at only 25–40% of the wt *Nras* level did not lead to any malignancies in heterozygous or homozygous mice [16]. Together this proves dose-dependency and cell type-dependency of NRAS induced malignancies and adds further complexity to the earlier depicted NRAS induced AML versus CMML question, since the transplantation of a higher number of *Nras^G12D/G12D^* myeloid cells accelerated but did not transform the CMML condition induced by *Nras^G12D/+^* cells [19].

Despite the examples of overexpression of wt *Nras* in human cancer and the large number of disease mouse models with deregulated *Nras* expression, only transgenic mice overexpressing wild type *Nras* under the mouse mammary tumor virus long terminal repeat are earlier described. These developed hyperplasias and malignant tumors in the tissues with the highest transgene expression [20]. We have recently developed a set of mouse models in which the murine leukemia virus Akv 1–99 long terminal repeat (LTR) was introduced within the *Nras* locus at the exact positions of previously identified retroviral integrations (B.B.G. et al, manuscript in preparation), [21].

Here we report on the early post natal pathology which is the consequence of an upregulated wt *Nras* expression from its endogenous locus, which is particularly increased in spleen. Shortly before death, animals present elevated levels of T-cells in spleen and myeloid cells in thymus, spleen, and blood. This immunological phenotype may help to understand the importance of NRAS in hematopoiesis and oncogenesis and add to the discussion on whether NRAS can act as initiator of abnormal cell growth eventually leading to cancer. The reported lethal phenotype has a complete penetrance within 3 weeks after birth, which indicates that the lethal condition does not require additional genetic events.

## Results

### Postnatal Lethal Phenotype of Homozygous *Nras^LTR9S^* Mice

**Figure 1:**
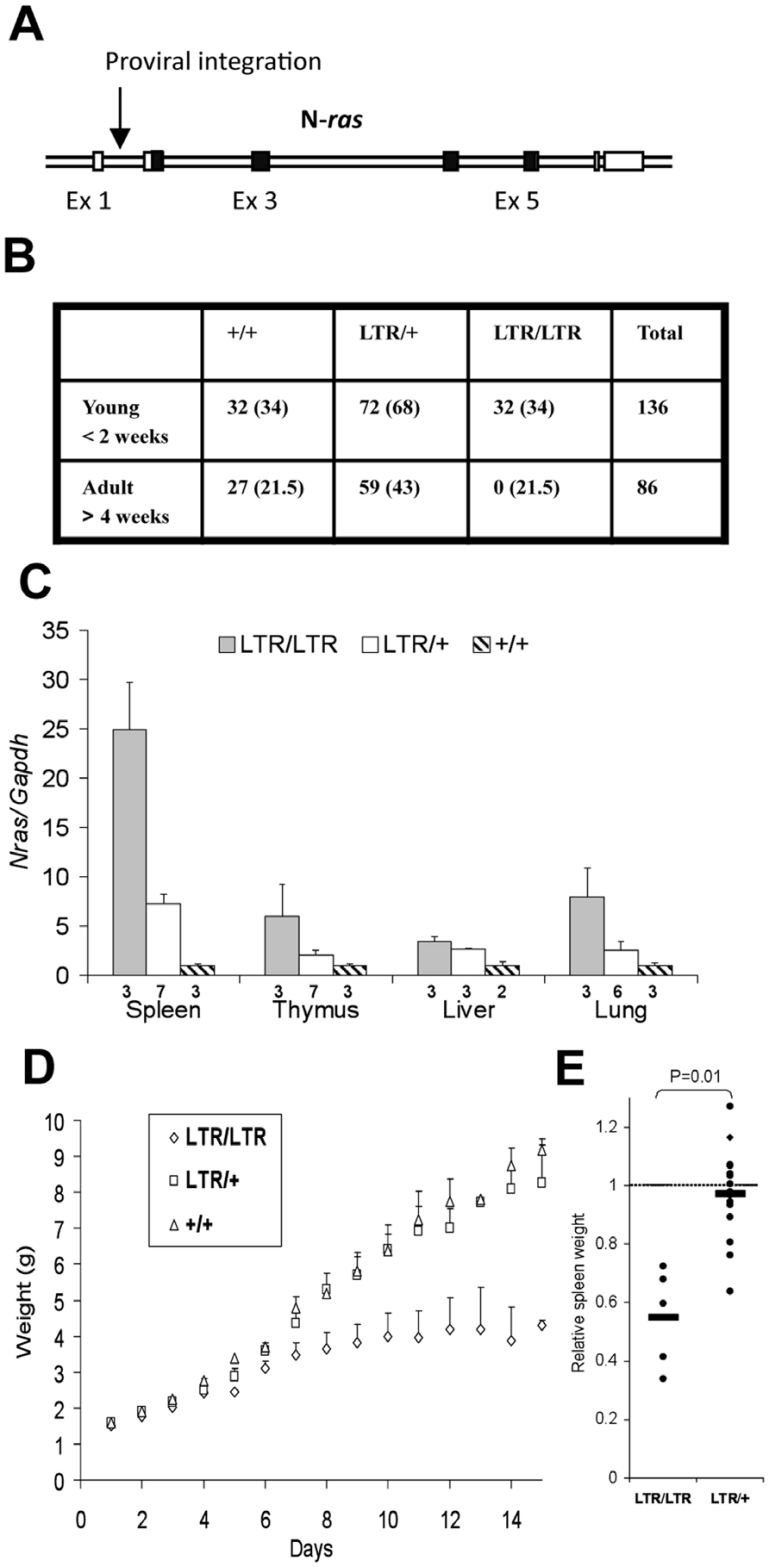
*Nras* overexpression resulted in weight reduction and early lethality. (A). LTR insertion at the proviral integration site in the *Nras* gene in transcriptional (sense) orientation. The LTR of Akv1–99 was inserted before nucleotide 1062 of the GenBank sequence no. L19607. Boxes indicate exons, coding regions in black. Exons and introns are not depicted in scale. Arrow indicates proviral integration. (B). Frequency of different genotypes in offspring from heterozygous matings. Numbers in parenthesis indicate the expected number of mice of each genotype. (C). Relative *Nras* expression in different tissues of young mice normalized to the expression of wild type tissues. *Gapdh* was used as internal standard. Error bars indicate standard deviation. Numbers under bars indicate numbers of analyzed mice. (D). Weight analysis of young mice. Homozygous animals showed a reduced weight gain after one week. Error bars indicate standard deviation. An average of 8 *LTR/LTR* mice, 12 *LTR/+* mice, and 5*+/+* mice are shown however, not all mice contribute to all time points. (E). Analysis of spleen weight relative to body weight. Bars show average of relative spleen weight normalized to wild type littermates. Relative spleen weight = Total spleen weight/total body weight. *LTR/LTR* mice have a lower relative spleen weight compared to wild type mice (n = 9).

*Nras^LTR9S^* mice harbored the LTR inserted before the first coding exon in the same transcriptional orientation as the *Nras* gene (Figure 1A). Sequence analysis confirmed the absence of mutations in the coding region of the targeted allele. Homozygous *Nras^LTR9S/LTR9S^* mice were born at the expected ratio but died before weaning (Figure 1B).

Q-RT-PCR analysis of young (10–12 days) and adult (40 days) hetero (*LTR/+*) and homozygous (*LTR/LTR*) *Nras^LTR9S^* mice revealed increased levels of *Nras* mRNA in all tissues analyzed. Compared to wild type littermates, most tissues showed a 5–7 fold or 3 fold upregulation of *Nras* expression in young *LTR/LTR* or *LTR/+* mice, respectively. As an exception, expression in spleen, the organ with the highest *Nras* upregulation, was increased 25 and 8 fold in *LTR/LTR* and *LTR/+* mice, respectively (Figure 1C). Young and adult *LTR/+* mice exhibited similar upregulated *Nras* mRNA levels compared to wild type littermates, resulting in higher NRAS protein levels as confirmed by Western blot analysis of spleen and thymus from adult mice (B.B.G. et al, manuscript in preparation). Analysis of single cell suspension of leukocytes from spleen of one litter including one *+/+*, five *LTR/+*, and three *LTR/LTR* mice revealed an upregulation of *Nras* mRNA at approximately the same level as whole spleen (23±0.4- and 9±0.2 fold in *LTR/LTR* and *LTR/+* mice, respectively), indicating that these cells contribute considerably to the *Nras* expression in the spleen.

At birth homozygous mice had the same weight as their littermates, but retarded after approximately one week, progressively losing weight during the following week. Some but not all heterozygous mice showed a transient reduction in body weight (Figure 1D), which was compensated before adulthood.

Even though all LRT/LTR mice died within a narrow time window, weight loss and disease development in homozygous mice differed among individual mice within the same litter. It was therefore necessary to analyze individual litters at different time points, when the most severely affected homozygous animals were near death. To compensate for this age difference, some results in this study were normalized within the litter. Unless otherwise stated, the age of the mice analyzed varied between 12 and 14 days. Homozygous mice showed a very pale liver suggesting severe dehydration, and weight analysis of the inner organs revealed a reduced spleen weight relative to body weight (Figure 1D).

### Granulocytosis in Homozygous Mice

**Figure 2:**
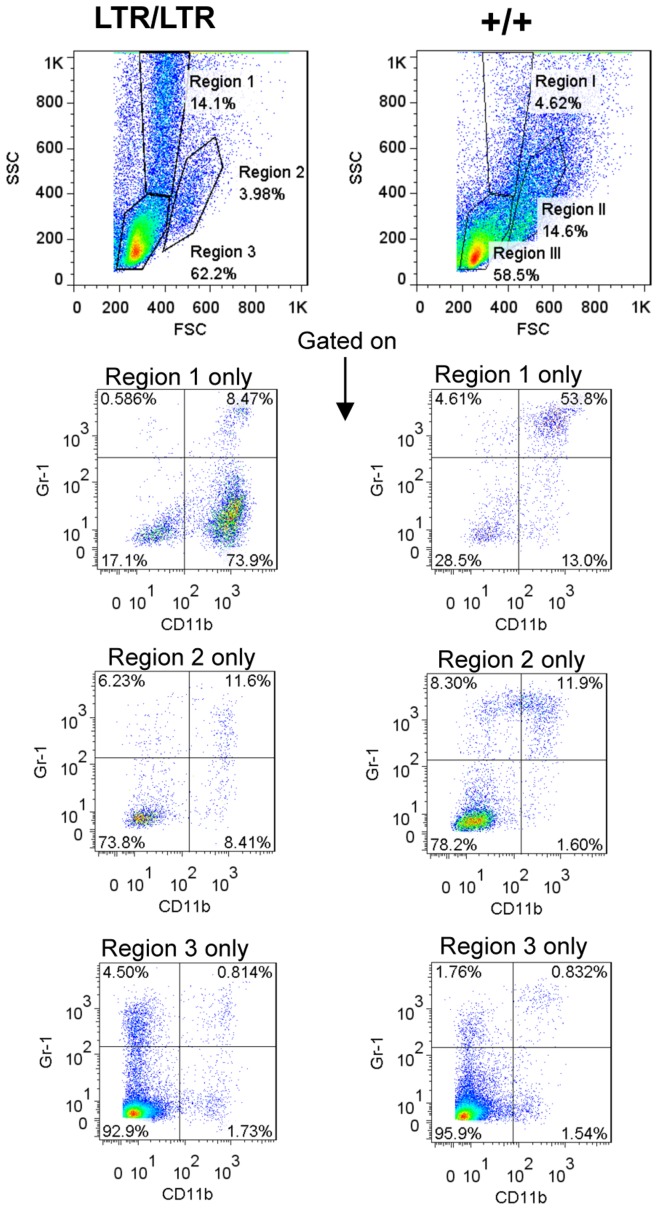
Flow cytometry analysis of the spleen of young *Nras^LTR9S^* mice. One representative analysis of a wild type and *LTR/LTR* mouse is shown. Debris and dead cells were excluded by applying a FSC threshold (channel 180). Significant upregulation of SSC high granular cells was detected in *LTR/LTR* mice (population is marked as region 1). A second population within the monocytic region is marked as region 2. The lymphocyte region is marked as region 3. Each region was analyzed for CD11b and Gr-1 stain.

**Figure 3:**
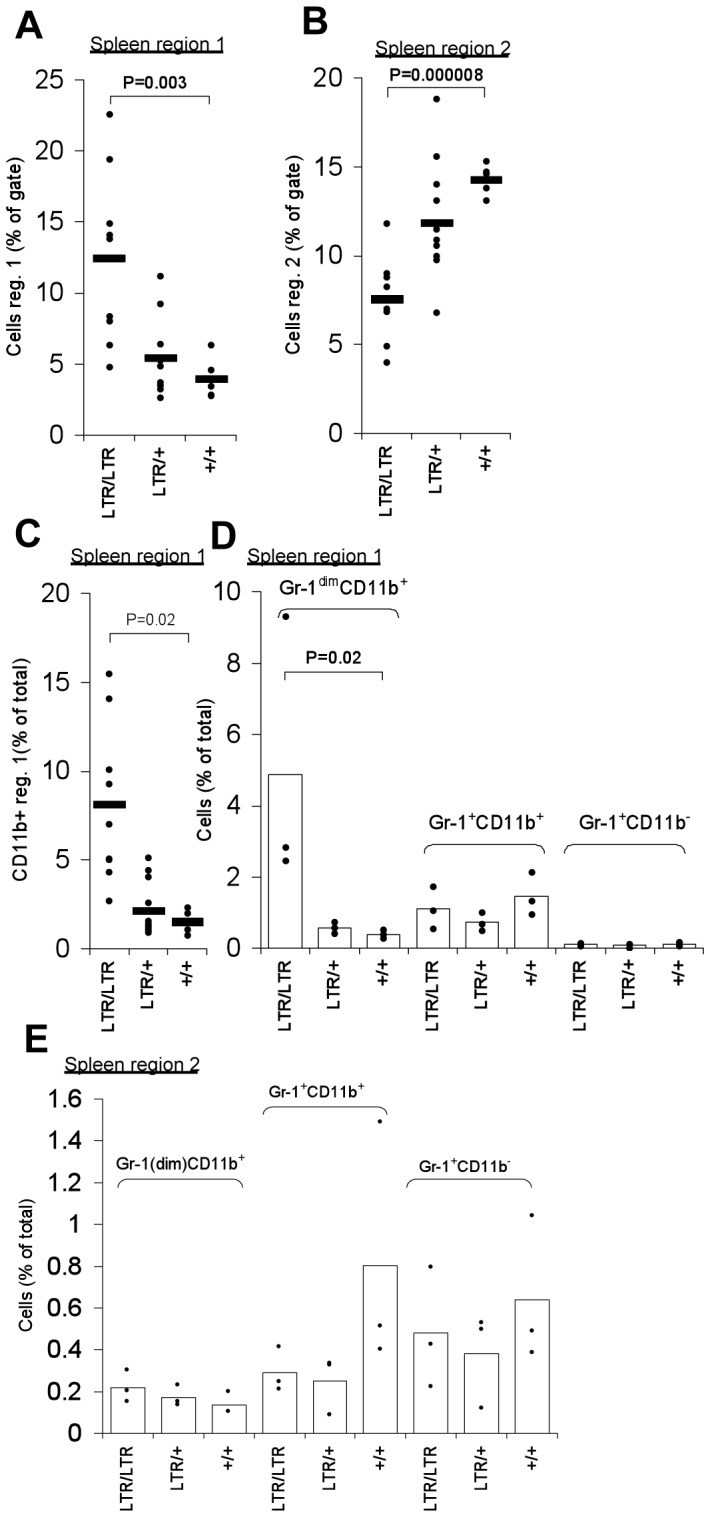
Flow cytometry analysis of myeloid cells in spleen of young *Nras^LTR9S^* mice. (A). Percentage of splenocytes within region 1 normalized to the gated population defined in Figure 2. Cell frequency was significantly higher in *LTR/LTR* mice compared to wt. (B). Percentage of splenocytes within region 2 normalized to the gated population defined in Figure 2. The population was significantly smaller in *LTR/LTR* mice compared to wt. (C). Percentage of splenocytes stained with CD11b within region 1 normalized to the total cell population. The CD11b^+^ population was significantly larger in *LTR/LTR* mice compared to wt. (D). Percentage of splenocytes stained with CD11b and Gr-1 within region 1 normalized to the total cell population. The Gr-1^dim^CD11b^+^ population was increased in *LTR/LTR* mice compared to wt. (E). Percentage of splenocytes stained with CD11b and Gr-1 within region 2 normalized to the total cell population. Dots represent individual mice.

Flow cytometry analysis of the spleen of young *Nras^LTR9S^* mice revealed an enlarged population with a high side scatter and the same localization as granulocytes. This population in the granulocytic region (GC region – region 1) increased from an average of 4.0% of the total viable cell number in wild type mice to 12.5% in *LTR/LTR* mice (Figures 2–3A). A statistically not significant intermediate phenotype was seen in heterozygous mice with an average population of 5.4% in region 1 (Figure 3A).

In contrast, the number of cells in region 2, where monocytes are normally localized, was decreased from an average of 14.3% in wild type mice to 7.57% in *LTR/LTR* mice. Again, heterozygous mice showed a statistically not significant intermediate phenotype (11.88%) (Figures 2 and 3B). As quantified for region 1 in Figure 3C, the population of CD11b^+^ cells increased in *LTR/LTR* mice.

Even though granulocytes are normally CD11b^+^ and Gr-1^+^ double positive, the majority of the increased population in region 1 was found to be CD11b^+^ and only weakly Gr-1 positive (Gr-1^dim^), indicating that immature granulocytes might constitute a major part of this increased population. Gr-1^+^CD11b^+^ and Gr-1^−^ CD11b^−^ cell numbers did not significantly differ among genotypes (Figure 3D). In region 2, the number of Gr-1^+^CD11b^+^ cells was clearly decreased in *LTR/LTR* mice (Figure 3E). Although not statistically significant, this indicates an altered lineage commitment from monocyte to granulocyte. However, in these mice the full development into granulocytes appeared interrupted or delayed as shown by the CD11b^+^Gr-1^dim^ stain pattern of the upregulated cell population in region 1. A minor statistically not significant upregulation of the CD11b^+^Gr-1^dim^ population was found in region 2 of *LTR/LTR* mice (Figure 3E). Notably, these cells were not a “spill over” from region 1 since they were distributed evenly within region 2. No consistent difference was found within region 3 in the spleen (data not shown).

**Figure 4:**
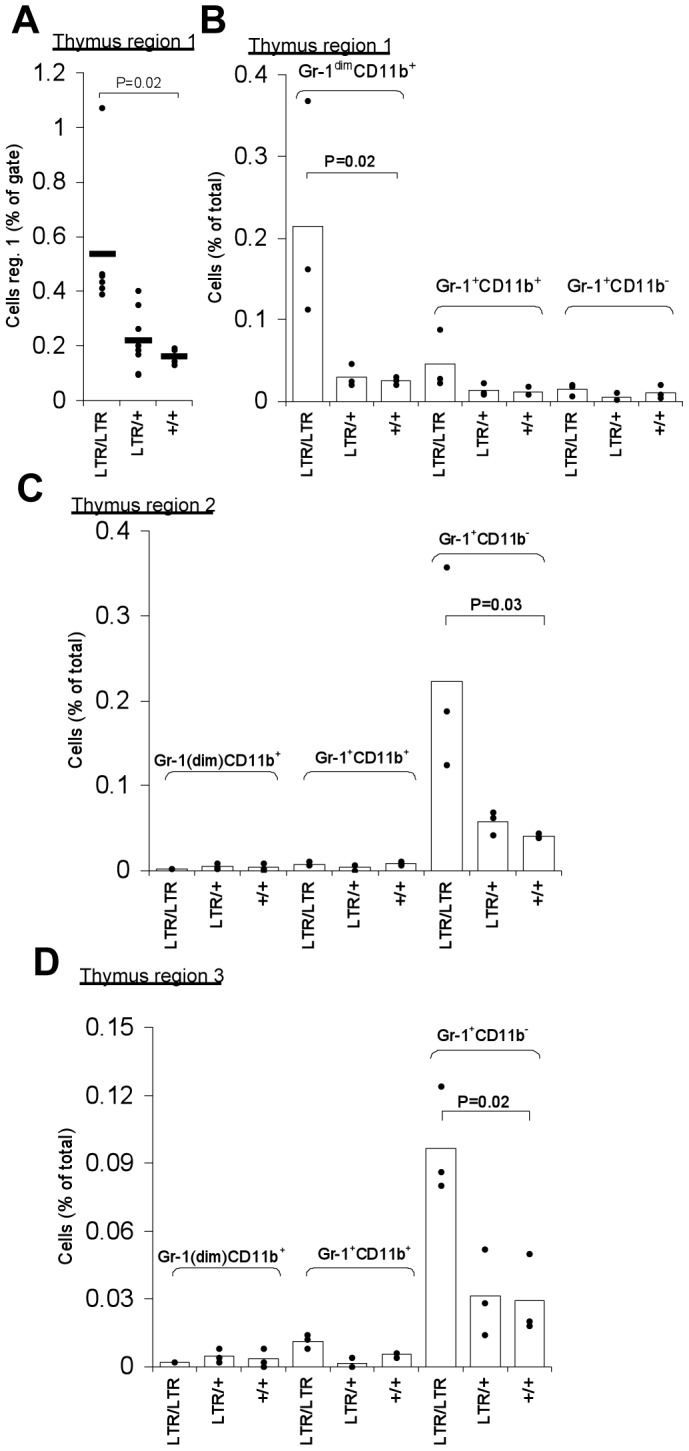
Flow cytometry analysis of myeloid cells in thymus of young *Nras^LTR9S^* mice. (A). Percentage of thymic cells within region 1 normalized to the population gated in the same way as shown for spleen data in Figure 2. Cell frequency was significantly higher in *LTR/LTR* mice compared to wt. (B). Percentage of thymic cells stained with CD11b and Gr-1 markers within region 1 normalized to the total cell population. The Gr-1^dim^CD11b^+^ population was upregulated in *LTR/LTR* mice compared to wt (data were log transformed to obtain variance homogeneity). (C). Percentage of thymic cells stained with CD11b and Gr-1 markers within region 2 normalized to the total cell population. The Gr-1^+^CD11b^−^ population was upregulated in *LTR/LTR* mice compared to wt (data were log transformed to obtain variance homogeneity). (D). Percentage of thymic cells stained with CD11b and Gr-1 markers within region 3 normalized to the total cell population. The Gr-1^+^CD11b^−^ population was upregulated in *LTR/LTR* mice compared to wt. Dots represent measures of individual mice.

In the thymus, region 1 consists of very few cells. Anyhow, the relative number of cells in this region was statistically significant increased in *LTR/LTR* mice (Figure 4A). This increase was again mainly due to Gr-1^dim^ CD11b^+^ positive cells (Figure 4B). The total cell number within region 2 and 3 was not significantly changed in the thymus but a statistically significant upregulation of Gr-1^+^CD11b^−^ cells was found in both region 2 and 3 (Figure 4C–D). This population might represent granulocytes, as infiltrating cells can shift their surface expression over time [22]. However, cases of Gr-1^+^CD11b^−^ cells that are CD3^+^ were previously reported, thus this population might also represent a rare subpopulation of T-cells [23].

**Figure 5:**
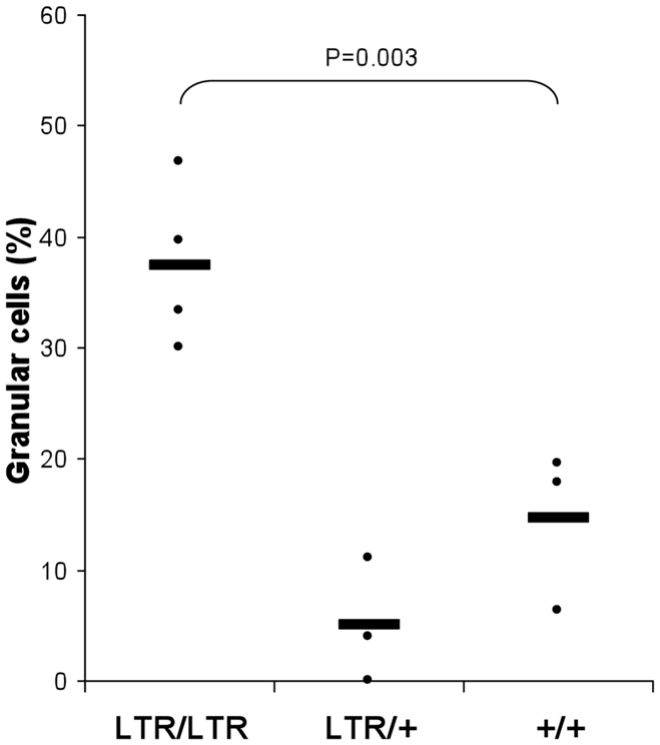
Analysis of granular cells in the blood of young *Nras^LTR9S^* mice. Relative numbers of granular and monocytic cells in blood smears. More granular cells and correspondingly less monocytic cells were found in *LTR/LTR* mice compared to wt (p<0.004).

To determine if the number of granular cells in the blood was increased we performed a Wright stain on blood smears from *Nras^LTR9S^* mice. Consistent with the larger population in the GC region observed by flow cytometry analysis a significantly higher number of granular cells was detected in the blood of homozygous mice (p<0.004) (Figure 5).

### T-cell Upregulation in the Spleen

**Figure 6:**
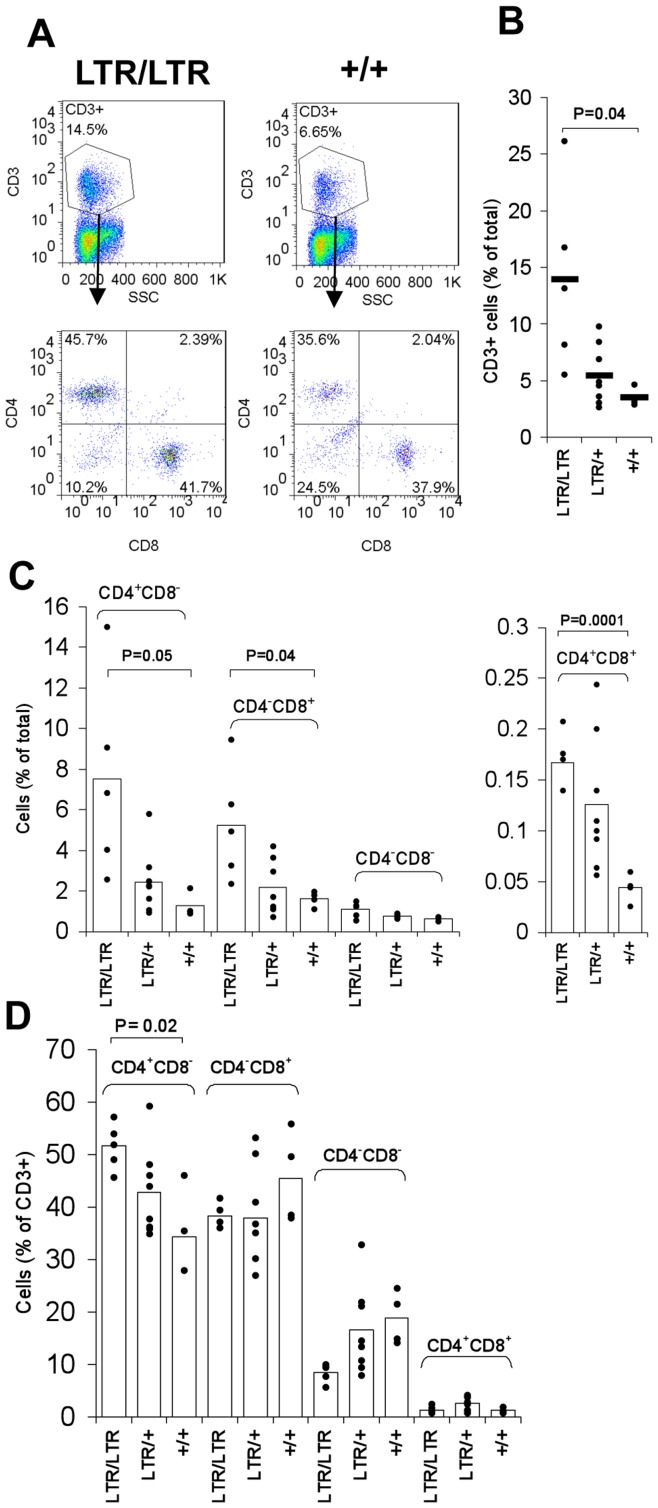
Flow cytometry analysis of T-cells in the spleen of young *Nras^LTR9S^* mice. (A). A representative analysis of one wild type and *LTR/LTR* mouse is shown. Lymphocytes were gated on and analyzed for CD3 expression as a marker of T-cells. The lymphocyte gate was set similar to region 3 shown in Figure 2. The T-cell population is more than doubled in homozygous *LTR/LTR* mice compared to wt. Subsequently the CD3^+^ population was analyzed for CD4 and CD8 expression. (B). Percentage of CD3^+^ T-cells in the spleen normalized to the total cell population. The frequency of CD3^+^ cells was statistically significantly upregulated in *LTR/LTR* mice compared to wt. (C). Overview of T-cell upregulation showing different subsets of T-cells normalized to the total cell population. A statistically significant increase of CD4^+^ cells, CD8^+^ cells and CD4^+^CD8^+^ cells was found in *LTR/LTR* mice compared to wt. (D) Overview of T-cell upregulation showing different subsets of T-cells normalized to the CD3^+^ cell population. A statistically significant increase of CD4^+^ cells was found in *LTR/LTR* mice compared to wt. Dots in (B), (C), and (D) represent results from individual mice.

Flow analysis of young homozygous mice showed an upregulation of CD3^+^ T-cells in the spleen from an average population size of 3.6% in wild type mice to 14.0% in *LTR/LTR* mice (Figures 6A–B). Heterozygous mice showed a statistically not significant intermediate increase to 5.5%. Within individual litters, homozygous mice that were more affected by the weight loss also showed a more severe upregulation of T-cells.

The upregulation of CD3^+^ cells in spleen of young mice was seen for both CD4^+^ single positive, and CD8^+^ single positive cells, and even within the small CD4^+^CD8^+^ double positive, and CD4^−^CD8^−^ double negative populations indicating that the upregulation is not specific for a single subset of T-cells (Figure 6C). However, the upregulation of double negative cells was not statistically significant. If compared within the CD3^+^ T-cell population, the frequency of CD4^+^ cells was furthermore significantly increased showing that there is a shift in the distribution of T-cell subpopulations (Figure 6D).

Analysis of T-cells in thymus showed a minor, not statistically significant upregulation. The fraction of single positive and double positive cells in thymus was unchanged, but a small increase was found among CD3^+^CD4^−^CD8^−^ cells (data not shown). However, comparison of the T-cell population size within litters showed a marked upregulation in two of the six analyzed homozygous mice, which both were significantly reduced in weight, indicating that the upregulation seen in thymus might be correlated with the progressing phenotype.

### *Gcsf* and *Gfi1* mRNA Increase in Homozygous *Nras* ^LTR9S^ Mice

**Figure 7:**
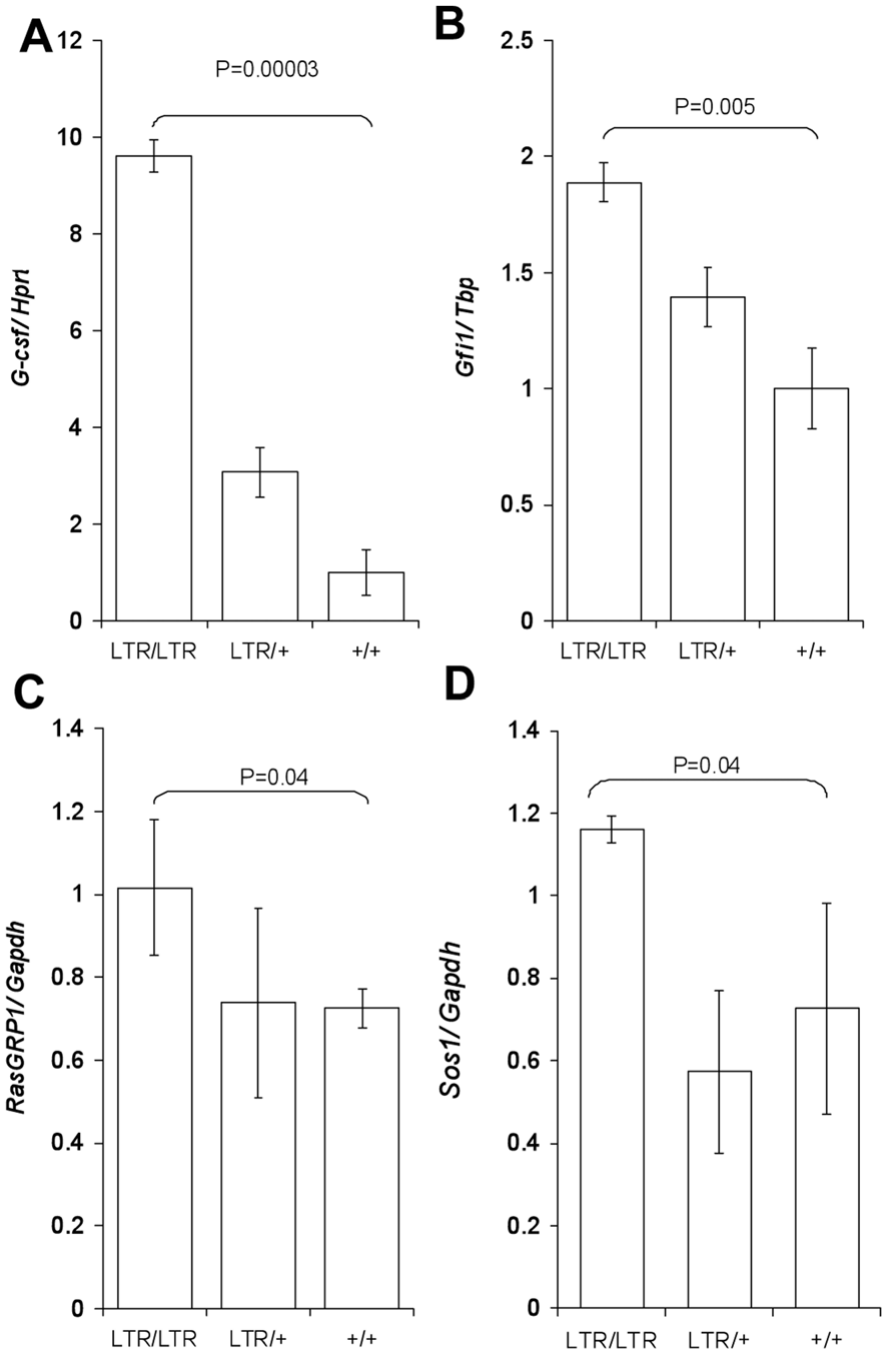
*Gscf* and *Gfi1* mRNAs were upregulated in young *Nras^LTR9S^* mice. (A). Q-RT-PCR quantification of *Gcsf* expression in spleen normalized to *Hprt* expression. Compared to wt *Gcsf* was upregulated in *LTR/LTR* spleen. (B). Q-RT-PCR quantification of *Gfi1* expression in spleen normalized to *Tbp* expression. Compared to wt *Gfi1* was upregulated in *LTR/LTR* spleen. (C). Q-RT-PCR quantification of *RasGRP1* expression normalized to *Gapdh* expression. Compared to wt *RasGRP1* was upregulated in *LTR/LTR* spleen. (D). Q-RT-PCR quantification of *Sos1* expression normalized to *Gapdh* expression. Compared to wt *Sos1* was upregulated in *LTR/LTR* spleen. For A and B three mice of each genotype were analyzed, for C and D, 3 LTR/LTR, 7 LTR/+, and 3+/+ mice were analyzed. Error bars indicate standard deviation.

The growth factor granulocyte colony stimulating factor (G-CSF) and the transcription factor growth factor independence1 (GFI1) are both involved in granulocyte differentiation [24], [25], [26]. In addition, GFI1 regulates G-CSF signaling through the RAS/MAPK/ERK pathway [27]. In order to determine the expression of both genes in spleen tissue of young *Nras^LTR9S^* mice, we performed q-RT-PCR analysis. We found that the level of *Gcsf* mRNA was increased 9.6 and 3.1 fold in homozygous and heterozygous mice, respectively (Figure 7A) and that the level of *Gfi1* mRNA was increased 1.9 and 1.4-fold in homozygous and heterozygous mice, respectively (Figure 7B). For both genes, the upregulation was statistically significant in *LTR/LTR* mice (p<0.001 and p<0.05, respectively).

### RasGEFs, *RasGRP1* and *Sos1* mRNA Increase Homozygous *Nras* ^LTR9S^ Mice

Due to the restricted expression of RasGRP1 found e.g. in T-cells and granulocyte progenitor cells we suspected this factor to be upregulated in homozygous *Nras* ^LTR9S^ spleen. Furthermore, we analyzed SOS1, which is an ubiquitously expressed exchange factor for RAS. Q-RT-PCR analysis showed a statistically significant (p<0.05) 1.4-fold upregulation of *RasGRP1* mRNA in the spleen of *LTR/LTR* mice. *Sos1* mRNA was likewise found to be upregulated in the spleen of *LTR/LTR* mice (1.6-fold; p<0.05).

## Discussion

With the aim to investigate the effect of deregulation of wt *Nras*, we have recently created several knock-in mouse models in which a murine leukemia virus Akv 1–99 LTR was inserted at different positions within the *Nras* locus (B.B.G. et al, manuscript in preparation). In each mouse line, a single LTR was introduced at the exact position of previously identified retroviral insertions known to result in B-cell lymphomas [21]. In one of the models, *Nras^LTR9S^*, the observed *Nras* upregulation resulted in decease of the homozygous animals before weaning. The mice suffered from significant weight loss and presented elevated levels of T-lymphocytes and myeloid cells within the spleen. This organ was reduced in the affected animals indicating altered cell proliferation, differentiation or survival. Furthermore, we found an increase of myeloid cells in thymus and blood, and an upregulation of *Gcsf* and *Gfi1* mRNA in spleen which likely contributes to the increase of myeloid cells. On the basis of flow cytometry and histology, we refer to this as granulocytosis. This is the fastest lethal phenotype likely caused solely by upregulation of wt *Nras*. The early lethality precludes the detection of a possible effect on lymphomagenesis of the inserted LTR as might have been expected form the underlying insertional mutagenesis study [21]. Transgenic mice with overexpression of wt *Nras* from a heterologouos promoter were previously reported to develope malignant tumors within one to a few months [20]. The constitutive overexpression of *Nras* in our model limits the possibility to directly compare the results to earlier studies on the effect of expression of mutated *Nras* in adult bone marrow cells. However the observation of an intermediate phenotype in heterozygous mice shows that the cellular expansion of T-cells and myeloid cells is dose dependent as reported earlier [14].

The early and uniform onset of the disease makes it very unlikely that a second event is required, even under the assumption that the *Nras* expression is already increased during embryonic development. However, because *Notch1* mutations are frequently associated with T-cell malignancies [16], we checked homozygous mice for the most frequent *Notch1* mutations and deletions in thymus of *Nras^LTR9S/LTR9S^* mice but found none (data not shown). The absence of *Notch1* alterations supports the conclusion that the increase of *Nras* expression is the primary cause of the observed lethal phenotype. A correlation between constitutively active NRAS and myeloid cells has been described in several mouse models [17], [13], [14], [18] and T-cell upregulation upon increased NRAS expression is consistent with the T-cell reduction found in the *Nras* KO mouse model [12] as well as the induced acute T-cell lymphoblastic leukemia/lymphoma in the *Nras^G12D/G12D^* bone marrow transplantation model [16].

Mutations causing constitutively active versions of RAS are often found in cancer and are likewise often used in transgenic mice and cell lines. In contrast, our model uses wild type *Nras* from the endogenous locus, and the NRAS protein still depends on physiological activation for its downstream signaling. The tissue distribution of the physiological activators may determine the final outcome of RAS signaling, since different activators of RAS might signal through specific downstream pathways. In T-cells, two RAS activator molecules RasGRP1 and SOS preferentially direct RAS signaling into different but possibly connected downstream pathways. Whereas SOS is ubiquitously expressed, RasGRP1 is only expressed in a limited number of tissues. In T-cells, studies have indicated that RasGRP1 is important for the initial triggering of RAS activation, whereafter SOS is activated by GTP-bound RAS starting a positive feed-back loop where both SOS and RasGRP1 are involved in RAS activation [28]. Consistent with this, we found *RasGRP1* mRNA levels to be upregulated in spleen of *LTR/LTR* mice. Furthermore, *Sos1* mRNA levels were found to be upregulated in spleen of *LTR/LTR* mice.

Consistent with the upregulation of *RasGRP1* mRNA levels, ERK induction as a result of RasGRP1 activity has been proposed to induce proliferation rather than differentiation [29], which would explain why we found upregulated levels of CD4^+^, CD8^+^, CD4^+^CD8^+^, and CD4^−^CD8^−^ cells in the homozygous *Nras^LTR9S^* mice. The fact that most cellular changes were found in the spleen where the most dramatic NRAS upregulation was seen likewise indicates a proliferative expansion rather than a peripheral phenotype caused by upregulation of the production of T-cells from the thymus.

Consistent with our observation of *Gfi1, G-csf, and RasGRP1* upregulation in the affected cells, GFI1 is found to regulate G-CSF signaling and subsequent granulocyte development through RasGRP1 and RAS. The ERK pathway is found to be impaired in *Gfi1* null cells and rescued by G-CSF. Our experiments revealed an upregulation of Gr-1(dim) cells consistent with the Gr-1(dim) staining observed in the RasGRP1 expressing cell population making it likely that this is an expansion of immature cells expressing the RasGRP1 activator. Together with the *Gfi1* mRNA upregulation this supports the interpretation that RasGRP1 is an important activator of the increased level of NRAS proteins, which then leads to ERK activation and subsequent proliferation rather than differentiation [27].

Likewise, Parikh et al. found the ERK pathway to be activated in most myeloid tumors induced by NRAS^G12D^ whereas the other major RAS signaling effector, AKT was only weakly activated [14]. The type of upstream activation of RAS could help determining the pathway of choice, similar to the finding that KRAS is the major regulator of cytokine dependent AKT-activation [30]. When stimulating *Nras^G12D^* -induced CMML tumor cells by GM-CSF, Wang et al. found the ERK pathway to be hyperactivated indicating that NRAS is important for cytokine dependent ERK-activation [18]. This is consistent with our observation of upregulation of the cytokine *Gcsf* mRNA. Furthermore, because RAS is activated by cytokines, *Gcsf* mRNA upregulation in homozygous and heterozygous *Nras^LTR9S^* mice suggests the existence of a positive auto-regulatory loop. This was earlier observed in human fibroblasts that express the oncogene version of *NRAS* where changes in the transcriptional level of IL-1, IL-6, G-CSF and GM-CSF were observed and the upregulation was found not to be caused by mutational events indicating a connection between RAS activity and cytokine production [31]. The existence of an auto-regulatory loop is further supported by the massive *Nras* upregulation in tissues of *LTR/LTR* mice which in most cases is increased more than twice compared to *LTR/+* mice. The question whether this regulatory feed-back works in an autocrine or paracrine manner still remains. Other cytokines may be involved, and it is possible that there is a link between T-cell and myeloid cell induction like e.g. the production of GM-CSF from T-cells possibly leading to granulocytosis [32].

In 2009 Morris et al. showed induction of cytotoxic T-cells after G-CSF administration in mice [33]. Together with the overexpression of *Gfi1* which is involved in T-cell proliferation [26], it might explain the increased number of T-lymphocytes detected in our homozygous mice.

Since granulocytes and monocytes are derived from common bone marrow progenitor cells, it is also tempting to speculate that we observe a reduction of monocytes in our analysis resulting from an altered cell fate decision [34]. Li et al. showed a higher expression of both *Nras* and *Kras* in early hematopetic and myeloid-restricted progenitor cells, which is consistent with a role of RAS in cell fate decision. Interestingly, *Kras* but not *Nras* is expressed at a higher level in mature granulocytes which might explain why we do not observe an increase in the mature granulocyte population [17].

The dramatic phenotype with a short latency shows that the cells are indeed able to activate the increased level of wild type NRAS expressed in these mice. The *Nras^LTR9S^* mouse might therefore be a valuable system to evaluate inhibitors of the NRAS pathway. Previous reports have shown a correlation between the level of RAS activity and the phenotype of the induced disease. Our model further supports this concept demonstrating that a moderate increase in *Nras* expression in heterozygous mice causes a mild phenotype, while high NRAS levels in homozygous mice are lethal. The short latency period to manifestation of the reported phenotype makes it highly likely that the manipulated *Nras* expression works as the primary and only genetic event causing the fatal condition in these *Nras^LTR9S^* mice.

## Materials and Methods

### Nras^LTR9S^ Mice

Knock-in animals were generated as described elsewhere (B.B.G. et al, manuscript in preparation). Shortly, homologous recombination of the *Nras* locus in CJ7 embryonic stem cells [35] was performed by a targeting vector consisting of the loxP-flanked PGK/Tn5 neomycin selection cassette and the Akv1–99 MLV viral LTR [36] (Figure 1A). Successfully targeted ES cells were injected into B6D2F2 blastocysts [37] and transgenic offspring mated with EIIa-Cre transgenic mice [38] to remove the PGK/Tn5 neomycin selection cassette. The resulting heterozygous *Nras^LTR9S/+^* mice were crossed to produce homozygous *Nras^LTR9S/LTR9S^* animals (named *LTR/+* and *LTR/LTR* in this paper). All experiments were performed with permission from the responsible local authorities.

### RNA Isolation

Total RNA was purified from frozen organs using TRIzol reagent (Invitrogen). Total RNA was reverse-transcribed using random hexamer primers with M-MLV reverse transcriptase (Invitrogen).

Determination of mRNA levels: Quantitative-real time-PCR studies were performed with Taqman hydrolysis probes (Applied Biosystems) in the Stratagene Mx3000 apparatus. Data were analyzed with the MxPro Software. For the transcripts of interest the *Nras* (Mm00477878_g1), *Gcsf* (Mm00438335_g1), *Gfi1* (Mm00515853_m1), *RasGRP1* (Mm01335285_m1), and *Sos1* (Mm01233256_m1) probes were utilized. *Gapdh* (4352932E), *Hprt1* (Mm00446968_m1) and *Tbp* (Mm00446973_m) were used for the quantification of the internal standards.

### Flow Cytometry Analysis

Monoclonal antibodies for CD3, CD8, Gr-1, CD11b, CD4 and blocking antibody (FcγIII/II receptor) were purchased from BD Biosciences. One million erythrocyte-lysed single cell suspensions from total spleen and thymus were incubated with 1 µg blocking antibody followed by 30 min incubation with surface marker monoclonal antibodies used in 2–3 color combinations for 30 min at 4°C, washed extensively with PBS plus 2% FCS, fixed with 1% paraformaldehyde, and analyzed by a single laser (488 nm) FACSCalibur (BD) equipped with standard optical filters and mirrors and set up using standard protocols and according to the manufacturer’s instructions. Standard “CELLQUEST Pro” acquisition analysis software (Becton Dickinson) was used for analysis. At least 5×10^4^ events within the primary gating (“Live Cell Gate”) based on cell scatter properties were acquired. Forward light scatter (FSC) and sideward light scatter (SSC) signals were expressed on a linear amplification scale. Fluorescence (FL) intensity signals were expressed as relative linear channels on a four-decade logarithmic scale. Irrelevant isotype-matched antibodies were used to determine background fluorescence. Unstained samples and samples including single-stain samples served as controls for subsequent software compensation using the “FlowJo” analysis software compensation module (Treestar). Antibody specificity including isotype controls was tested on normal spleen and thymus mouse tissue due to limited amount of tissue from the affected mice.

Fluorochrome conjugated antibodies were used in two panels: Anti-CD8-PerCP-Cy5.5 and anti-CD4-FITC were used in combination with anti-CD3-phycoeryrthrin (CD3-PE) as a pan T-cell marker to distinguish between different T-cell differentiation stages. Anti-Gr-1 (PE) and anti-CD11b-fluorescein isothiocyanate (CD11b-FITC) were used in combination to identify myeloid cells. Non-stained samples were run as negative controls.

### Blood Stains

Blood smears were stained with Wright stain (Sigma Aldrich) for 15 seconds according to the manufacturer’s recommendation.

### Statistical Analysis

For expression analysis of *RasGRP1* and *Sos1*, Q-RT-PCR experiments were repeated and data were analyzed by the unpaired *t* test. For all other data, paired Student’s *t* test was used to test for statistical significance. P-values less than 0.05 were considered statistically significant. Some data sets were log-transformed to obtain variance homogeneity.
